# Bilateral Optic Disc Edema in a Patient with Lead Poisoning

**DOI:** 10.18502/jovr.v14i4.5465

**Published:** 2019-10-24

**Authors:** Kaveh Abri Aghdam, Amin Zand, Mostafa Soltan Sanjari

**Affiliations:** ^1^Eye Research Center, The Five Senses Institute, Rassoul Akram Hospital, Iran University of Medical Sciences, Tehran, Iran

**Keywords:** Lead Poisoning, Opium Dependence, Optic Disc Edema, Paresthesia

## Abstract

**Purpose:**

Heavy metals, such as lead can cause optic neuropathy. Optic disc neuropathy due to lead intoxication has previously been reported. We report a rare case of lead toxicity-induced optic neuropathy presenting with bilateral hemorrhagic optic disc swelling.

**Case Report:**

The patient was a 42-year-old man with a history of chronic oral opium use, who had a gradually progressing blurred vision in both eyes over 40 days, with ataxia, paresthesia, and a toxic level of serum lead. He had been treated with lead chelators for lead poisoning. His color vision was impaired in both eyes. Humphrey's visual field test revealed double arcuate scotoma with enlargement of the blind spot. Funduscopy revealed bilateral optic disc swelling, which was confirmed on optical coherence tomography and fluorescein angiography.

**Conclusion:**

In cases of optic disc edema, a comprehensive history should be taken to detect the cause. Further, in cases of chronic oral opium use, lead toxicity should be considered.

##  INTRODUCTION

Optic disc swelling or edema can be unilateral or bilateral. Bilateral disc edema can occur due to increased intracranial pressure (ICP), inflammatory processes, toxic agents, nutritional deficit or hypertensive emergencies.^[1]^

Toxic optic neuropathies are a group of disorders defined as visual impairment caused by damage to the optic nerve by a toxin. This condition often presents as a progressive, painless bilateral vision decline with central or cecocentral scotoma and reduced color vision.^[2]^

Heavy metals are a group of toxic agents that can cause toxic optic neuropathy. Lead poisoning is a common cause of metal toxicity. There are several sources of lead exposure, such as paint, gasoline, cosmetic products, and batteries. Absorption of lead occurs via the respiratory and gastrointestinal tracts.^[3]^ The most common symptom in lead toxicity is systemic hypertension. The neurotoxic effects of lead on the human body are clinically well-known, including optic neuropathy.^[4]^ The retina, as a part of the central nervous system, is sensitive to lead toxicity.^[5]^ Lead accumulation in the retinal pigment epithelium (RPE) can cause apoptosis of the photoreceptors, especially rods, which can be detected as decreased sensitivity and amplitudes of dark-adapted electroretinogram (ERG) waves. This condition may present with peripheral retinal pigmentary changes.^[5,6,7]^ The aim of this report was to describe bilateral optic neuropathy, a rare ocular manifestation of lead toxicity, in a patient with opium addiction, which was evaluated using clinical examinations, laboratory tests, and multimodal imaging.

##  CASE REPORT

**Figure 1:**
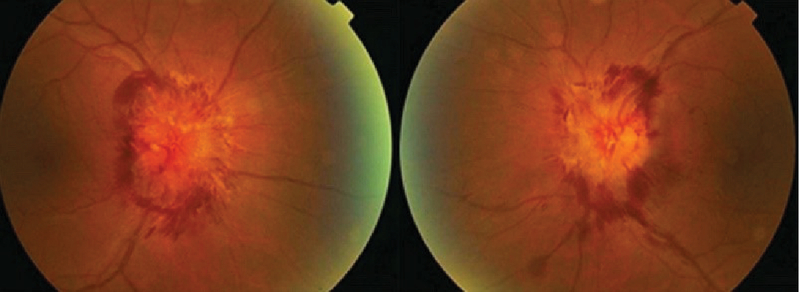
Fundus photographs show significant hyperemia and edema of both optic discs.

**Figure 2:**
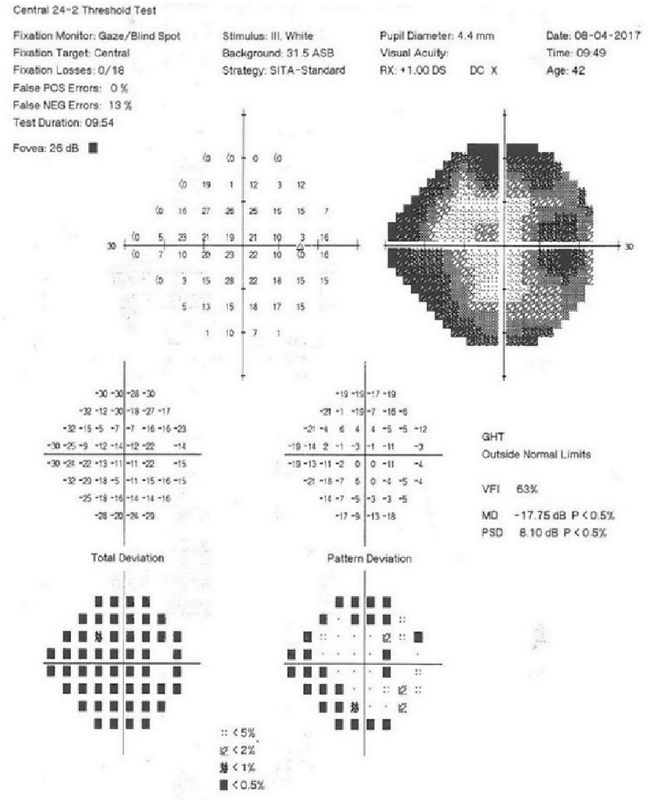
Humphrey's visual field test of the right eye (SITA standard 24-2) shows double arcuate scotoma with enlargement of the blind spot and severe generalized depression.

**Figure 3:**
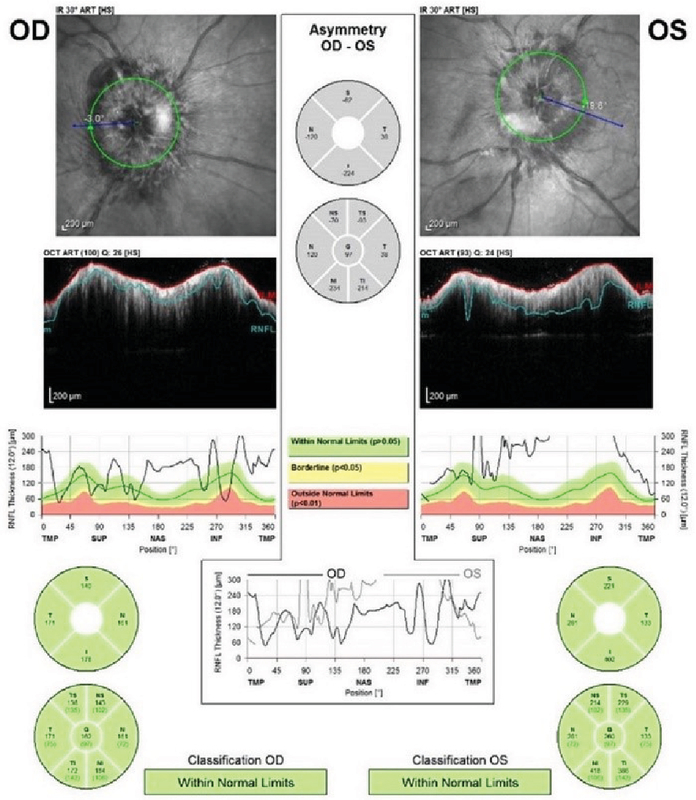
Spectral-domain optical coherence tomography shows increased reticular nerve fiber layer thickness in all four quadrants in both eyes, suggestive of disc edema.

**Figure 4:**
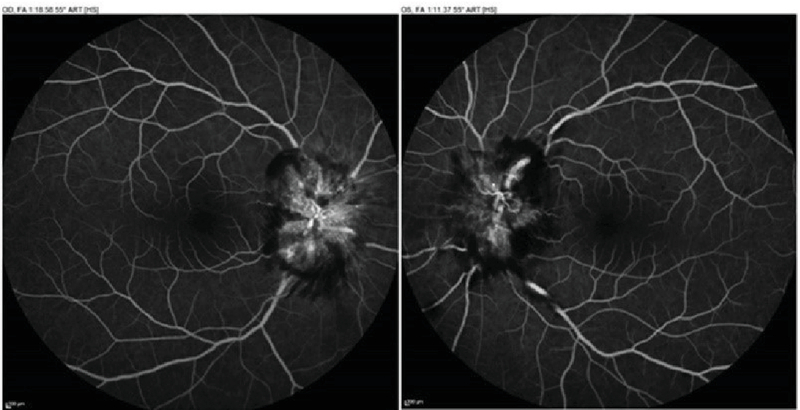
Fluorescein angiography shows disc leakage in both eyes, suggestive of disc edema.

A 42-year-old male shopkeeper was referred to the Neuro-ophthalmology Clinic at the Rassoul Akram Hospital, Tehran, Iran with blurred vision in both eyes. The patient complained of gradual progression of bilateral vision decline, ataxia, and paresthesia of the lower extremities over the past 40 days. Results of brain magnetic resonance imaging (MRI) with and without contrast were normal, and the ICP was within normal limits. The patient's medical records indicated that the serum lead level was 164 µg/dL (normal upper limit: 20 µg/dL). One month before the referral, he had been treated with intravenous sodium calcium edetate (disodium calcium ethylenediaminetetraacetic acid [EDTA]) for five days by a neurologist. Subsequently, succimer (2,3-dimercaptosuccinic acid) was orally administered for two weeks. A week later, his neurological symptoms improved, and the lead level in his serum dropped to 36 µg/dL. Before treatment, the patient had not undergone eye evaluation. Shortly after the completion of chelation therapy, he came to our neuro-ophthalmology clinic for the evaluation of persistent blurred vision in both eyes, although he reported subjective improvement in visual acuity after chelation therapy. His medical and family histories were unremarkable. His blood pressure was 115/80 mm Hg. He had used oral opium for over 10 years but did not drink alcohol. With the Snellen chart, his best-corrected visual acuity was 5/10 at a distance of 6 m in the right eye and “count fingers” at a distance of 3 m in the left eye. There was no vision improvement in the pinhole test in either eye. His pupillary reactions were normal in both eyes, with a relative afferent defect in the left eye. Extraocular motility was intact in both eyes. The intraocular pressure was 14 mm Hg in both eyes on Goldmann applanation tonometry. The Ishihara test scores were 9/14 and 1/14 for the right and left eyes, respectively. Slit-lamp examination was unremarkable. His dilated fundus exam was significant for hyperemia, hemorrhage, and edema of the retinal nerve fiber layer surrounding both optic discs [Figure 1]. Optic nerve pallor was not present. The macula, peripheral vascular structures, and retinal periphery were unremarkable in both eyes. The Humphrey visual field test showed double arcuate scotoma with enlargement of the blind spot and severe generalized depression of the right eye [Figure 2]. The visual field of the left eye was severely depressed, and the patient could not undergo perimetry. Peripapillary optical coherence tomography (OCT) demonstrated increased thickness of the retinal nerve fiber layer (RNFL) in all four quadrants of both eyes [Figure 3]. Fluorescein angiography detected disc leakage in both eyes [Figure 4].

The patient underwent lead toxicity treatment with chelating agents (EDTA and succimer) and showed a good response with a decreased serum lead level. He was advised to attend follow-up visits to monitor the resolution of optic neuropathy with clinical and paraclinical tests. However, the patient did not return for follow-up visits to detect clinical and/or paraclinical progression or regression of the disease.

##  DISCUSSION

Sources of lead include old paint, old plumbing, glass-making, dust exposure, battery burning, and soldering.^[5]^ However, our patient was not exposed to any of these sources or risks. He was a chronic oral opium user, and recently, the opium used by him contained lead as a chemical impurity. Lead is occasionally added to opium products to increase its weight for higher profits.^[8]^

Lead poisoning can cause systemic hypertension, which can cause hypertensive optic neuropathy.^[5]^ However, the patient had no history of systemic hypertension. According to the patient's medical records at the time of presentation, his blood pressure was 115/80 mm Hg with no evidence of accelerated or malignant hypertension. Moreover, cotton-wool spots, hard exudates, Elschnig spots, and Siegrist streaks, which are indicative of hypertensive chorioretinopathy were absent [Figures 1 and 4].

Toxic optic neuropathies typically present with a gradually progressive, bilaterally symmetric, painless vision loss affecting the central vision. Poisoning with heavy metals, such as lead, can cause toxic optic neuropathy.^[9,10]^ Lead exposure has a toxic effect on the macular, choroidal, and RNFLs, which causes visual deficits.^[11]^ Our patient had a progressive, painless bilateral visual acuity decrement, color vision impairment, and visual field defects.

Bilateral optic disc neuropathy with lead intoxication has previously been reported. Baghdassarian reported a 49-year-old male paint-shop worker who had bilateral optic neuropathy and showed laboratory evidence of lead poisoning.^[4]^ Therefore, our patient with bilateral hemorrhagic optic disc edema, as indicated on funduscopy and imaging, is a rare case.

Heavy metals, including lead, can accumulate in human pigmented ocular tissues, particularly in RPE and the choroid, resulting in decreased sensitivity and amplitude of the a- and b-waves of the dark-adapted ERG.^[5]^ Lead exposure causes retinal degeneration with selective loss of rods and bipolar cells.^[12]^ This is due to apoptosis of the photoreceptor cells.^[7]^ Lead can also cause disorders in calcium metabolism of the photoreceptors.^[7]^ Rod degeneration in patients with lead retinal toxicity can present with peripheral retinal pigmentary alterations.^[6]^ Our patient refused to undergo ERG for further investigations. However, funduscopy did not reveal any retinal peripheral abnormalities.

Although neuroimaging shows normal results in toxic optic neuropathy, it is almost always indicated to rule out space-occupying lesions. The most appropriate imaging modality is MRI of the optic nerves and chiasm with and without gadolinium enhancement.^[2]^ Brain MRI of this patient was unremarkable, with no enhancement of the optic nerves, which did not favor the diagnosis of optic neuritis.

The treatment of optic neuropathy due to lead poisoning includes removing the sources of lead exposure and chelation with EDTA and succimer. Chelating agents for lead poisoning can reduce the serum lead level and improve the clinical and paraclinical features of toxic optic neuropathy due to systemic lead poisoning.^[5]^ For example, Baghdassarian reported a patient with bilateral optic neuropathy because of systemic lead poisoning in whom the visual acuity significantly improved and visual field scotoma decreased after treatment with the lead-chelator D-penicillamine and subsequent

serum lead level reduction.^[4]^ Our patient had been treated with EDTA, and subsequently with succimer. After the treatment, the serum lead level decreased from 164 to 36 µg/dL. The neurological symptoms disappeared, and visual acuity in both eyes improved subjectively.

A limitation of this study is that we did not measure the macular ganglion cell layer thickness with OCT; it could help with the evaluation of cell loss in cases of optic disc edema. Papilledema is defined as optic disc edema due to increased ICP and should be differentiated from papillitis. The lumbar puncture in this patient showed that ICP was within normal limits, which ruled out papilledema. Therefore, we considered bilateral hemorrhagic optic disc swelling due to lead toxicity. Neurological symptoms of lead toxicity, normal ICP (which ruled out idiopathic intracranial hypertension), visual acuity impairment, positive response to chelation therapy, and other clinical and paraclinical evaluations confirmed that the underlying cause was lead toxicity.

In cases of optic disc edema, physicians should take a comprehensive history to detect the etiology. In cases of chronic oral opium use, lead toxicity should be considered, as lead is occasionally added to the product.

##  Financial Support and Sponsorship

Nil.

##  Conflicts of Interest

There is no conflict of interest.
